# “You lose confidence in yourself, I think, a little bit…” A qualitative study exploring the perceptions and experiences of the barriers and facilitators to wellbeing in women in work over 60 years old

**DOI:** 10.3389/fsoc.2026.1781316

**Published:** 2026-07-21

**Authors:** Clare Edge, Giulia Bacci

**Affiliations:** 1 School of Health & Society, University of Salford, Salford, United Kingdom; 2 Department of Psychology, University of Turin, Turin, Italy

**Keywords:** ageing, intersectionality, wellbeing, women, workplace

## Abstract

**Introduction:**

Life stress accumulation across the life course disproportionately negatively impacts wellbeing, cognition, and health in ageing women, yet there is a paucity of research exploring the perceptions of ageing women in work.

**Methods:**

This study draws upon unique data to provide an understanding through illuminating the intersectional wellbeing of gendered ageing in ageing women in the workplace in UK focusing on lower-paid roles in the Northwest of England. Semi-structured interviews were used to explore the perceptions of older women’s wellbeing in an opportunity sample of (*n* = 19) of women aged 60 and over with a focus on those in lower-paid and part time roles (although *n* = 4 were in senior roles). Reflexive thematic analysis was used to analyze the data.

**Results:**

There were two central themes: Barriers to Wellbeing and Facilitators to Wellbeing. Barriers to wellbeing encompassed the following subthemes: (1) the Taboo of Womanhood: the limiting nature of being an ageing woman as a barrier to wellbeing; (2) Feeling of Invisibility: intergenerational barriers to wellbeing; (3) The workplace environment: prolonged standing, and physical and emotional strain nature of role as a barrier to wellbeing; (4) Negative self-perceptions of growing old: health deteriorations, mortality perceptions, and lack of energy; (5) Financial factors as a barrier to ageing women’s workplace wellbeing. Whereas facilitators to wellbeing were encompassed in the following subthemes: (1) The value of older women in work as a facilitator to wellbeing; (2) Support from line manager as a facilitator to wellbeing; (3) Rejecting the label/stereotype as ‘older’ as a facilitator to wellbeing.

**Discussion:**

Workplace policies and practices should actively challenge the taboo surrounding women’s wellbeing, value older women in work, and provide flexible, supportive environments and management that enable open conversations and respond to women’s changing needs across the life course.

## Introduction

Globally, women are working for longer, with women’s labor force participation reaching record levels of 66% in 2023. The largest increases are evident among women aged 55–64 years (Organisation for Economic Co-operation and Development (OECD), 2024). The Office for National Statistics (ONS) (2024) data show that women in the 55–64 years old group had the highest levels of sickness absence, with absence due to stress being most likely in this group (Office for National Statistics (ONS), 2024). In the United Kingdom, women face gender pension gaps, pay gaps, gender care gaps, and gender health gaps. These are important to consider based on the structural inequalities women face across the life course in the context of wellbeing and extended working life.

Whilst in the United Kingdom (UK), women’s pension age has been harmonized, research demonstrates women disproportionately extend their working life compared to men and are affected by inequalities, including health inequalities, over the course of their lives more than men (Finch, 2014). Looking specifically at lower-paid workers, LaBond et al. (2022), focusing on blue-collar bus drivers, highlight that women are over-represented in lower-paid roles characterized by reduced autonomy and financial precarity. For many women, the need to remain in paid employment into older age reflects economic necessity rather than preference, reinforcing the importance of sustaining health and wellbeing at work. Despite this, women in lower-paid roles remain under-represented in research on later-life working, which has tended to prioritize professional or managerial occupational groups.

Whilst the term “older worker” typically refers to individuals aged 55 years and over (EU-OSHA – European Agency for Safety and Health at Work, 2016), definitions vary, with some European frameworks categorizing those aged 50 + as older workers (Kooij et al., 2008; Zaidi, 2008). The Office for National Statistics (Office for National Statistics (ONS), 2024) data show that women aged 55–64 report the highest levels of sickness absence in the UK workforce, with stress-related absence particularly prevalent in this age group (Office for National Statistics (ONS), 2024). Although the state pension age for women and men has been harmonized (Department for Work and Pensions (DWP), 2014), research demonstrates that women are disproportionately more likely than men to extend their working lives. Working into later life and extended working life is, therefore, embedded within gendered life-course structures.

Later working life should also be examined in relation to unpaid labor. Women are significantly more likely than men to combine paid work with extensive unpaid domestic and caregiving responsibilities, resulting in what has been widely described as a “double burden.” Gendered divisions of unpaid labor persist across the life course (Doyal, 1995; Payne, 2008). Across the European Union, women aged 55 and older undertake an average of 18 additional hours of unpaid work per week (262 min per day), compared with 5 h per week (141 min per day) for men (Organisation for Economic Co-operation and Development (OECD), 2024; Parent-Thirion et al., 2007). Despite longstanding recommendations from the International Labour Organisation, organizational and policy recognition of family responsibilities continues to prioritize younger women, rendering older women’s unpaid labor largely invisible (Ghosheh et al., 2006).

The consequences of unpaid labor, undertaken alongside paid work, are substantial for both physical and mental health. Caring for elderly dependents can result in physical strain comparable to that encountered by paid caregivers, such as nurses, including the risk of back injuries from lifting and moving individuals (Doyal and Daykin, 1999; Walters et al., 2002). The physical demands of caring for young children, including grandchildren, are less often recognized, yet they can also affect the health of older women. Tasks such as lifting, carrying, and bending can contribute to back pain and musculoskeletal disorders (Alcouffe et al., 1999). In addition to physical health impacts, unpaid caregiving alongside paid work is associated with increased stress, emotional exhaustion, isolation, and poorer mental health outcomes (Ervin et al., 2022).

To date, there have been reviews conducted on the menopause (Atkinson et al., 2021; Dennis and Hobson, 2023; Hardy et al., 2018; Jack et al., 2016), the taboo of women’s health (the three M’s: menstruation, maternity, menopause) (Grandey et al., 2020), extended working life (Edge et al., 2017; Ni Léime and Ogg, 2019), and retirement and health impacts (van der Heide et al., 2013). These reviews have found that limited qualitative studies have explored the intersection of ageing and gender, and where they do exist, they focus on EWL, retirement or menopause, or identity rather than holistic wellbeing.

There is, however, growing research interest around intersectional factors around women’s wellbeing and menopausal symptoms in the workplace in the UK and increasing public awareness around this topic (Beck and Brewis, 2024). Yet, Ni Léime et al. (2017) highlight how the UK extended working life policy does not consider the gendered dimensions of unpaid labor and the emotional tolls associated with these across the life course, hence the need to focus on women nearing retirement age. Critiques of UK extended working life policy further highlight its neoliberal foundations and its lack of sensitivity to regional and socio-economic inequalities (Macnicol, 2015; Lain et al., 2019; Lain et al., 2022). This highlights the importance of a regional focus exploring regions such as the North West and North East of England where there are persistent inequalities (Office for National Statistics (ONS), 2024).

The present study addresses this gap by focusing on the North West of England, a region characterized by persistent health, income and employment inequalities (Office for National Statistics (ONS), 2024), and the geographical area in which the study institution is situated. As Vickerstaff (2017) argues, understanding extended working life requires an interdisciplinary approach that integrates occupational health, human resource management (HRM), and sociological perspectives.

### Literature review: occupational health perspectives on ageing women’s workplace wellbeing

The focus of this article is on ageing women’s workplace wellbeing and the barriers and facilitators to these. It is important to consider the occupational and human resource management aspects related to health and wellbeing, as well as the gendered ageing perspectives to workplace wellbeing.

A seminal occupational medicine review (Payne and Doyal, 2010) into ageing women’s health highlighted the need to focus on gender and ageing in the workplace due to a range of risk factors for women, including the risk of musculoskeletal issues, mental ill health, and menopause. Recent evidence demonstrates that these conditions are interconnected across the life course rather than discrete, with menopause often exacerbating musculoskeletal pain and mental health difficulties (Brown et al., 2024; Heikkinen et al., 2022).

Ageing women report more work-related stress than ageing men (Organisation for Economic Co-operation and Development (OECD), 2024), and there is a key trend in stress accumulation across the life course, which disproportionately and negatively impacts wellbeing, cognition, and health in older women than older men (Miller et al., 2022). Women in later working life consistently report higher levels of work-related stress than men of the same age (Organisation for Economic Co-operation and Development (OECD), 2024). Stress is shown to accumulate across the life course, disproportionately affecting women’s wellbeing, cognitive functioning, and long-term health outcomes in older age (Miller et al., 2022). Across the life course, women may also experience a range of hormone-related health conditions, including premenstrual syndrome, fertility challenges and endometriosis, all of which influence workplace experiences and long-term wellbeing (Sang et al., 2021).

Other biological drivers relating to ageing as a woman across the life course include predispositions to a range of ill health-related issues, including being more predisposed osteoporosis, arthritis and autoimmune diseases, anemia, and thyroid conditions, among a range of other health issues (Overstreet et al., 2023). This contributes to the well-documented “gender health paradox,” whereby women live longer than men but spend a greater proportion of later life living with illness or disability and show higher levels of sickness absence whilst in paid employment (Office for National Statistics (ONS), 2024).

### Occupational wellbeing and human resource management perspectives

In HRM literature, recent attention has been emphasized on the link between multi-faceted occupational wellbeing (comprising happiness or psychological, health, or physical and relational or social wellbeing) and so-called ‘high-performance work practices’, i.e., those which impact positively on organizational performance. However, this has been demonstrated to be a highly complex picture in terms of how these HRM practices underpin wellbeing. For instance, some practices, such as performance-related pay, are shown to increase elements of wellbeing (e.g., happiness), whilst impacting negatively on health-related wellbeing (Guerci et al., 2019, 2022; Guest, 2017, 2024).

Warr’s (2007, 1987) “vitamin model” identifies 12 features associated with positive employee wellbeing, including autonomy, skill use, environmental clarity, social contact, equity, security, and supportive supervision. Notably, equity, defined as justice within organizations and fairness in societal relations (Warr, 2011), is particularly salient when examining gendered experiences of ageing at work. Wellbeing is increasingly seen as important for organizational performance, hence the need for organizations to invest in the wellbeing of their employees (see, for example, Guest, 2024).

### Gendered ageing perspectives on workplace wellbeing

From a gendered ageing perspective, Gullette (2004) argues that individuals are “aged by culture,” through narratives that frame ageing as decline and diminished value. For women, ageing is shaped by the intersection of ageism and sexism, often referred to as “sexageism.” Theoretical frameworks such as gender performativity (Butler, 2002), gender role congruity theory (Eagly and Karau, 2002), social identity complexity (Roccas and Brewer, 2002), ideal worker norms (Reid, 2015; Steffan, 2021; Riach, 2025), and intersectionality (Crenshaw Crenshaw, 1989) provide insights into how organizational norms shape ageing women’s wellbeing.

Riach (2025) (p.19) highlights that for women growing older in the workplace is particularly challenging for the ageing self because *“gender relations set up unattainable and unsteady trajectories for those who are beyond the dominant embodied form of a masculine, white corporate worker.”* In fact, Riach (2025) highlights the notion of hypervisible and problematic or invisible in respect of ageing women in the workplace. Similarly, Taylor et al. (2021) highlights the need to recognize older women in the workforce. These dynamics operate unevenly across occupational status, with women in lower-paid roles experiencing intensified disadvantage due to reduced power and heightened exposure to expectations of concealing age-related decline (Barrett, 2022; Westwood, 2023).

Across the life course, women encounter persistent taboos surrounding menstruation, maternity, and menopause (Grandey et al., 2020). Ideal worker norms have been shown to advantage constant availability and uninterrupted career trajectories, disproportionately disadvantaging women during both early-career maternity and later-life menopause transitions (Bolino et al., 2013; Padavic et al., 2020). Research demonstrates that menopausal women often avoid disclosure due to anticipated stigma, particularly in male-dominated workplaces, limiting access to workplace adjustments (Griffiths et al., 2013; Hickey et al., 2017). Even senior women report feeling marginalized during menopause, describing their bodies as “out of place” within organizations (Mavin and Grandy, 2016). Disclosure of symptoms and their menopausal experiences often left women feeling some reputation damage and a loss of perception of authority because they were no longer seen as ‘reliable workers’ (Lorber and Moore, 2002). Additionally, women in work as they age are shown to be impacted by stereotype threat (Manzi et al., 2021), which in turn impacts their performance, but the removal of the stereotype (i.e., that older women are not as competent as older men) improves performance.

## Significance and originality of the study

This study makes an original contribution by focusing on the intersection of gendered ageing and the perceptions of ageing women’s workplace wellbeing. The current study answers calls for further research exploring the intersectional experiences of ageing women and responds to the growing need to understand the experiences of ageing women in lower-paid roles (LaBond et al., 2022; Ni Léime et al., 2017). This research is of key international significance because of the lack of research exploring the perceptions and experiences of women in lower-paid roles and their wellbeing in work, as well as the intersection of ageing and gender, specifically. Research has highlighted the need to illuminate the voices of older women because they are shown to experience invisibility as they age (Westwood, 2023; Riach, 2025). The study also has clear policy and practice significance by focusing on a region facing inequalities.

### Research question

Taken together, this literature highlights a significant gap in understanding how ageing, gender, occupational status, and wellbeing intersect, particularly for women in lower-paid roles. Therefore, the research question is as follows:

*What are the barriers and facilitators to workplace wellbeing among women over 60, with a focus on lower-paid roles?*

## Methods

### Design

Semi-structured interviews carried out in person (*n* = 17) and online (*n* = 2) were used to explore the experiences and perceptions of older women’s wellbeing in an opportunity sample of (*n* = 19) older women aged 60 and over, with a focus on those in lower-paid and part-time roles, although *n* = 4 were in senior roles. A minimum of 15 participants is regarded as acceptable for a reflective thematic analysis (Braun and Clarke, 2013), and there were finite resources, whereby the study was advertised in local workplaces on a poster and included an offer of a thank you of £20 for taking part. Semi-structured interviews were chosen because they enable a deeper understanding of the unique perceptions and subjective experiences of individuals, whilst framing the topic of interviews within the parameters of the research question. A social constructivist paradigm using an interpretivist lens was adopted, whereby the analytical lens explored the narratives of participants and how they constructed and perceived their experiences of wellbeing at work.

### Participants and data collection

The interview questions explored older women’s current workplace and role, perceptions of being a woman and an older woman in the workplace, experiences of the barriers and facilitators to wellbeing at work and any experiences of stereotypes, and prejudice and stigma associated with being an older woman in the workplace. An example question being ‘can you tell me about your perceptions and experiences of the barriers to workplace wellbeing?’ [prompts included workplace factors, a lack of support from colleagues]. The whole dataset was used in this analysis, but another analysis was carried out in a separate study using a subset of the data focusing on research questions around older women’s identity and self-beliefs (Edge and Bacci, 2024), distinct from this study, which focuses on wellbeing.

The reason women over 60 years old have been chosen is that this age group will be approaching/have reached or exceeded the statutory pension age, which is important because of the recent changes to state pension age globally. This study used an opportunity sample recruiting from organizations across Greater Manchester, in the north of England where older women in lower-paid part-time roles were found, e.g., ‘5 Cs’: catering, cleaning, caring, clerical, and cashiering. However, several women also came forward who were in roles classed as professional roles (*n* = 4). All participants were assigned a participant number, and their role classification can be found in Table 1.

**Table 1:** Participant characteristics: job role.

Job role type	*N*	%
Customer service, secretary, or receptionist role	8	42
Supervisor and senior manager role	4	21
Community Freelance Artist & Writer	1	5
Social care role	1	5
Health care worker or public health role	3	16
Education role	2	11
Community and volunteer worker	1	5

### Data analysis

Braun and Clarke’s (2006, 2022) reflexive thematic analysis was used to analyze the data. The data were analyzed using a primarily data-led (inductive) approach, but theoretical constructs such as Warr’s vitamin analogy were also applied to some of the coding using a more deductive approach. The six steps of reflexive analysis were applied by the primary researcher [Clare Edge], with coding and themes/subthemes checked by [Giulia Bacci]. Braun and Clarke (2022) argue that theoretical saturation is not congruent with reflexive thematic analysis, where codes and themes are actively generated through researcher interpretation, and data collection and coding were not determined by saturation. Instead, these processes were guided by the richness and depth of the data, with attention to the point at which new analytic insights became more limited.

### Reflexivity statement

The research team comprised individuals with backgrounds in Work and Organizational Psychology [Giulia Bacci] and Community Psychology and Wellness at Work [Clare Edge], with experience in qualitative research employing reflexive thematic analysis. We recognize that our professional positions and prior assumptions may shape data collection and interpretation. To address this, reflexive practices were embedded throughout the research process, including regular team discussions, reflexive notes, and critical examination of emerging interpretations to ensure that the findings were grounded in participants’ accounts rather than researcher preconceptions.

Throughout the process, I [Clare Edge], as the lead researcher, employed in-depth reflexivity, engaging on my position in the subjective process as a woman who might be perceived as comparably younger and in a higher status position. I was keen to break down boundaries at the beginning of the interview by introducing myself and my research background to try to reduce any power dynamics that may exist. I was also aware of my position as a ‘younger’ (39/40 years old at the time) woman, and this had a bearing on the findings in that the taboo and invisibility element of the data resonated with me as under-discussed outside of the interviews but were frequently mentioned by women in the study and stood out as interesting. Finally, the interview process itself was impacted by my reflexivity based on my perception of the negative connotations surrounding the word ‘older’, which were articulated by several women who did not ‘feel older’, and therefore, I altered the terminology from older woman to woman over 60 years (Table 2).

**Table 2:** Main themes and sub-themes.

Theme 1: Barriers to Women’s Workplace Wellbeing	Theme 2: Facilitators to Women’s Workplace Wellbeing
The taboo of Womanhood: *a lack of flexibility and needs under-recognised- health and wellbeing needs and caring responsibilities as a barrier to wellbeing*	The value of ageing women in work as a facilitator to wellbeing
Feeling of Invisibility: *Intergenerational Barriers to Wellbeing*	
Negative perceptions of growing old: *health deteriorations, mortality perceptions and lack of energy*	Support from line manager as a facilitator to wellbeing
The workplace environment: prolonged standing, strain and nature of role as a barrier to wellbeing	Rejecting the label/stereotype as ‘older’ as a facilitator to wellbeing
Financial factors as a barrier to ageing women’s workplace wellbeing	

## Findings

The two key themes and seven subthemes identified in relation to the research question reflect a range of views across the dataset in women aged 60 and over in the workplace centering around two themes: (1) Barriers to Wellbeing and (2) Facilitators to Wellbeing.

### Theme 1: Barriers to Women’s Workplace Wellbeing

(i) The taboo of Womanhood: a lack of flexibility and needs under-recognized- health and wellbeing needs and caring responsibilities as a barrier to wellbeing

There was a perception across the participants that women felt their physiology, hormonal changes, and the menopause were not accommodated and therefore had negative impacts on women in work, such as having a lack of confidence and their progression being stunted. Interviewees felt that women’s health needs as they age including menstruation, menopause, bladder issues, musculoskeletal problems and impacts on mental health, and the vast majority felt they were hardly recognized and a ‘taboo’. There were contrasting views of how the workplace should accommodate these. One woman described how she felt the system should accommodate women retiring earlier than men because their physiology is inherently different.

*“…when you are going through the menopause…a lack of oestrogen in your body can do a lot of things, because I was reading a menopause book as well, and some of the things that it was describing, at one time I thought I was going really daft…I went to the doctors and said I think I’m losing my marbles…I think sometimes as well, going through that sort of thing, I think that stopped me from progressing more than I probably would have done, because you lose confidence in yourself, I think, a little bit”* [Participant 3, 64 years old, Customer Service Role, Large Public Sector Organisation].

However, a contrasting view from another participant was displayed in a neoliberal notion (Lain et al., 2022; Steffan, 2021) of ‘just get on with it’ but also describing that she had been ‘quite lucky’ with the menopause. She described that as a younger woman, she needed to work part-time to cope with heavy periods and described younger colleagues having musculoskeletal issues that were not considered in working patterns with prolonged standing.

Participant 1 described having bladder problems whilst at work, but not having this accommodated in working patterns (not being able to leave the phone stations), and another participant described asking for direct accommodations for bladder weaknesses (by being asked to be stationed near an accessible toilet) but not being considered.

*“The barriers* [to wellbeing]*, is the aches and pains of getting older, I do not think it’s recognised at all. It’s like, you know, suck it and just get on with it. There’s all sorts of - it should not stop us from working, just the problems that comes with age, like you have bladder problems, but you are not allowed to be - you are not allowed to be off your phone that many times. Well, I’m sorry. I’ve got to, you have got to* [use the toilet].” [Participant 1, 64 years old, Receptionist role, Large Public Sector Organisation].

*“When I asked about being put at a desk near the accessible toilet, I was asked, ‘Do you not wear a pad?’ I said, ‘What’s it got to do with you?. the accessible toilet is right in front of me’. She said to me, ‘I’m sure you could make it to the disabled toilet in* [name of building].*’ I said, ‘Do not tell me what I can do, because I cannot.’…and so, I am still off sick”* [Participant 2, 65 years old, Customer Service Role, Large Public Sector Organisation].

Women felt that it was difficult to speak about taboo health issues as a woman in general, but this was particularly so with male managers, and many women felt there was a need for training to enable these conversations, so both men and women can discuss these topics openly in the workplace, offering the correct support/workplace adjustments. Participant 17 described as a woman having the ‘additional kind of burden of responsibility for caring and everything’ and went on to describe the organization tasks women take on compared to men such as arranging holidays and paying bills. This was a common theme across the interviews, and the level of the burden of care across the interviewees in women aged 60 and over in work was overwhelming. All of these factors were felt to have taken a toll on women in work and negatively impacted their wellbeing, underlining the need for flexibility and accommodations for these needs. Participant 6 described her life since having extra caring responsibilities, which became heightened over the COVID-19 pandemic due to mental health issues of her son, and this had a significant negative impact on her wellbeing as she described no longer doing yoga, walks, and so on that were positive activities for her wellbeing.

*“Apart from that, sleep when I can get it, the occasional walk. I loved, before any of this started to happen* [caring for her sick adult son]*, before 2006, I used to get up in the morning, I used to do yoga. I loved it. Made me feel great. I felt all fizzy before I went to work. I felt really good. Not done yoga for years. Cannot get the space.”* [Participant 6, 65 years old, Medical Secretary, Large Public Sector Organisation].

(ii) Feeling of Invisibility: *Intergenerational Barriers to Wellbeing*

Across the interviews, there was a perception of intergenerational barriers between young and older women, whereby they felt younger people prefer younger staff and older workers are not seen as managers. There was also a perception of having a lack of energy with age (both self-compared to younger workers and from younger members of the team); a feeling of impatience for lack of understanding of technology, whereby some older women workers described being spoken to with a lack of respect by younger staff. Other women talked about feeling like an outsider, invisible, and not included, and this subtheme was an overwhelming element of this theme. There was a perception of youth being valued more than age and a lack of support from managers to progress compared to younger colleagues. However, women described that they wanted to keep updated with skills as they felt the chance to work for longer is a choice they should be able to take rather than feeling forced into retirement. There was a feeling that technology was a key intergenerational factor acting as a barrier, whereby older women felt they were not as ‘tech savvy’ as younger workers who saw them as the ‘granny’, ‘mum’, and ‘tea lady’ and this was apparent across the interviews but particularly for those in lower-paid roles. Women felt that they were seen by the younger generation as ‘old-fashioned’, ‘daft’, ‘dizzy’, ‘stupid’, and ‘grumpy’ and ultimately described feeling invisible.

*“…time moves on, and IT* [Information Technology] *moves on, and we have got self-scan now. We’ve got smart shop. We’ve got all sorts of things, and we have had to learn that. You’ve got Microsoft Edge. All that wasn’t there when I first started, so to say that old people cannot learn with the technology, it’s whether they want to learn. It’s there. Some people go, ‘Oh, I do not know how to do it.’ It’s like if you rang up an electrician and they go, ‘Oh, have you taken the plug out?’ and all that, and you think yes, I have… Yes, I think sometimes they do perceive that older women are not in with whatever is new.”* [Participant 5, 64 years old, Customer Service Role, Large Private Sector Retail Organisation].

There was an overwhelming feeling of invisibility with age as a woman across the interviews relating to various self-perceptions, which seemed to make them feel inferior or lower status compared to their younger counterparts:

*“You feel invisible as you get older…people just laugh at you really”* [Participant 16, Community and Volunteer Worker 70 years old, Voluntary Sector Organisation].

*“I just want to say that you get invisible as a woman after a certain age”* [Participant 19, Education Role, 65 years old, Large Public Sector Organisation].

Participant 12 went on to describe the lack of opportunities as an older woman with an implicit assumption from her manager that as an older woman she would not be looking to develop or enhance her skillset in respect of promotion opportunities compared with younger women:

*“A lot of the younger ones seem to have been put on training courses and their manager was sending them - they have all had a rejig of their job plans and so they are all doing courses to enhance their career progression. My manager did not even look at me and he just assumed that I did not want to do it.”* [Participant 12, Health Care Role, 60 years old, Large Public Sector Organisation].

Participant 8 reflected that although she perceived negative connotations associated with being seen as an ‘older woman’ by some members of her team, as discussed in the previous theme, she simultaneously described feeling valued by her director because of her age and experience. She noted that assumptions that older women are resistant to change can be countered by recognizing their accumulated experiential knowledge, particularly their understanding of what works and what does not over time.

*“…my director, I think, really does value me, and I think she does take what I have to say seriously and does give feedback positively…It’s about people understanding more roundly what the issues are rather than just jumping in at the deep end…”* [Participant 8, 61 years old, Senior Manager Role, Large Public Sector Organisation].

Participant 14 highlighted a feeling of having fewer opportunities for promotion with age and a feeling of older women not having long left in their working life from colleagues:

*“I think you’d feel that you get less chance of being promoted as you are getting older, because they’ll think, ‘She’s not going to be in there that long anyway, and we’ll have to retrain again.’”* [Participant 14, 64 years old, Customer Service Role, Large Public Sector Organisation].

*“I think they assume that my age group have not got managerial skills, without a doubt.”* [Participant 1, 64 years old, Receptionist role, Large Public Sector Organisation].

(iii) Negative perceptions of growing old: health deteriorations, mortality perceptions and lack of energy

There was a perception across a range of women in both lower-paid and senior roles that growing older as a working woman led to a lack of energy, along with health deteriorations and a perception of morality. There was a feeling that there is a significant amount of prejudice and discrimination present, and this has a negative impact on wellbeing:

*“You are you visualized as slower, both physically and mentally.”* [Participant 14, 68 Years Old, Public Sector Manager Role, Large Public Sector Organisation].

*“Just not being able to properly cope, that they are* [older women] a *bit past it, not as speedy as the youngsters. You have to be able to keep up, and think quickly, and if you are a little bit more like, ‘Oh, do you want a cup of tea, love? Sit down.’…”* [Participant 9, 64 years old, Social Care Role, Large Public Sector Organisation].

Participant 8 relayed she compared herself to younger members of the team and in doing so recognized differences in energy and ambition compared to her younger sense of self:

*“The only thing I find quite difficult in my* [team] *is that I’m probably one of the oldest, if not the oldest which is quite a challenge, really, when you are working with the majority young people. I think, also, sometimes you tend to think, well, I was like that 30 years ago. You know when you see new lots of energy and lots of ambition and think, yes, I remember when I used to have the energy to do that!”* [Participant 8, 61 years old, Senior Manager Role, Large Public Sector Organisation].

Participants 8, 9, and 14 noted this perception but directed the construction of this feeling externally at the comparisons to younger people. It was more nuanced in that it refers to social norms and perceptions of colleagues in relation to older women rather than an internalized viewpoint *per se* that they agreed with. Participant nine also highlighted that she felt some women do go along with the ‘stereotype’ of needing to take break as an older woman and needing special compensation or using this to avoid being as productive as younger women. Whereas participant 11 described her own fears around her mortality as she gets older as did a range of other participants:

*“I’ve also got this at the back of my mind, my mother was so young-looking and so fit and healthy, and she did not have to work, but at age 68 she had a major stroke from which she never recovered. So, I think that’s in the back of your mind as well”* [Participant 11, 64 years old, Customer Service Role, Large Public Sector Organisation].

(iv) The workplace environment: prolonged standing, strain, and nature of role as a barrier to wellbeing

Several women described the nature of the work in the customer service as having a negative impact on their wellbeing as they were growing older in terms of struggling with prolonged standing. They felt that the workplace should accommodate their needs as they grew older by providing adjustments such as a chair or adapting the role in other ways to accommodate health needs. Participant 7 described the perception of others that she should not be doing heavy lifting rather than herself describing the negative impacts of heavy lifting on her own wellbeing indicating a perception from colleagues that older women are not capable.

*“It’s standing up all the time, you are on your feet all the time. The only time you sit down is when you go on your break. The rest of the time you stand up. I remember when I first went to* [name of organisation]*, they had tills there with seats. Not anymore. No. No, there’s no sitting down. On your feet till you break, and that’s it…. but my work mates are great.* [they say] *‘You should not be doing that, do not you pick that up. That’s too heavy for you.’ ….” [Participant 7, 80 years old, Customer Service Role, Private Sector Retail Organisation].*

*“I cannot do things physically like running up the stairs like I used to…I do not wanna do anymore lugging about…the younger ones should be doing more of this, not me…I’d run up and down stairs with the full box of stuff, and now I’m thinking I cannot do that. I do not wanna do that. So that’s my well being there.”* [Participant 15, 64 years old, Customer Service Role, Private Sector Organisation].

A number of participants reflected that wellbeing is not supported because the work still needs to be done: Physical health can be adjusted for, but mental wellbeing is not considered:

*“I just think it’s empty words really, because on the other hand, you have still got to work. You still got to meet that deadline. Still got to do this, still got to do that. You get a chair if you have got a bad back. You might get an adaptation to your keyboard, or whatever, or your computer screen, but from a mental health wellbeing, I do not think that’s very good at all.”* [Participant 10, 65 years old, Education Role, Large Public Sector Organisation].

(v) Financial factors as a barrier to ageing women’s workplace wellbeing

The final barrier around financial planning into retirement exacerbated by the impacts of the cost of living articulated by the vast majority of participants when considering the impact of both pension changes in the UK and the cost-of-living crisis.

*“I think if I’d had the option of retiring sooner, I think I would have done. I’ve got a friend of mine that is a couple of years older than me and we always said about when we retire, we’ll be going out and we’ll be trying to find a bit of a life, and then you find out that you have got a few more years after her to go. Since she retired, I’m thinking to myself, well, that could have been me, I could have been retired now, and maybe with my grandson as well, taking on more responsibility with him.”* [Participant 3, 64 years old, Customer Service Role, Large Public Sector Organisation].

*“…it causes me great stress because I worry about money. I always have done, and I always will do. Now we are seeing the mortgage go up, we are seeing food prices, fuel. Everything is going up, but our wages aren’t, not in comparison. It’s hideous because I keep thinking, oh my gosh, if this carries on, how are we going to afford the mortgage? How are we going to afford food?”* [Participant 4, 60 years old, Public Health Role for Local Authority].

A range of women described the negative impact of pension changes on their wellbeing. Women described feeling shocked and upset that they needed to work for longer and were missing out on enjoying what they had imagined from an early age would be their retirement. One woman relayed being part of the Women Against State Pension Increases (WASPI) march of women against state pension increases in Parliament.

*“Well, to, to be honest, I did not know about it, and I joined that WASPI* [Women Against State Pension Increases] *thing. Yeah, on a march in London… But I do feel really disgruntled about that. I’ve been working since I was eleven… I stood outside the Houses of Parliament*.*”* [Participant 18, 60 years old, Health Care Worker, Large Public Sector Organisation].

Whilst another woman described the feeling of resent toward policy change, how it was implemented and the impact this had on her wellbeing.

*“I think because you always expected you’d be finishing work at 60, it’s a heck of a shock. I mean I cannot… I did actually receive a letter before, you know, my time because I know like these WASPI women, it’s all geared towards that they have not had this letter. But I think you do begin to be a bit resentful that they have added a full six years on…*.” [Participant 11, 64 years old, Customer Service Role, Large Public Sector Organisation].

### Theme 2: Facilitators to Women’s Workplace Wellbeing

(i) The value of ageing women in work as a facilitator to wellbeing

Many women acknowledged the value they bring as ageing women and felt their experience was a competitive advantage compared to younger workers. One woman described having felt overwhelmed as a younger woman managing the finances of the household and being older, she could see that she could have done things differently. Many women felt they have better social skills and customer service skills as an older woman compared to younger workers.

*“And that and that sort of comes with experience as well. You’re doing the job for 15 years. So, you have got that. And also, the passion behind your motivation for doing the job, which comes from your own experience. Yes. So, I am very passionate about it and that’s something that you cannot teach.”* [Participant 18, 60 years old, Health Care Worker, Large Public Sector Organisation].

*“But there have been other occasions where I feel that being the older person has given me more kudos because I like I say…because I was the person who they could say, well, why do they do that and when did they change that? And I would know because I’ve been there a long time, and I had the experience.”* [Participant 14, 68 Years Old, Public Sector Manager Role, Large Public Sector Organisation].

*“I was like the manager of the family with the finances and everything and it was just all too much* [as a younger woman]. *So, that was a big thing because I’d never gone through it before, but then when you get older and you see people going through it you think, yes, I know, but if only I’d have known now, I should have done this and I should have done that.”* [Participant 6, 65 years old, Medical Secretary, Large Public Sector Organisation].

*“And as you get older, you do tend to be more chatty, if that makes sense, because you are focused on more in the interaction, the social interaction of what makes people happy.”* [Participant 15, 64 years old, Customer Service Role, Private Sector Organisation].

(ii) Support from line manager as a facilitator to wellbeing

Various women reflected on the importance of a good line manager and the importance of being open in that relationship and the notion of the manager standing up for the worker and valuing their contributions. Women reflected on previous relationships being better than current relationships, and several women described currently having a supportive manager who accommodated her needs and valued their experience.

*“I mean she was really good. She was like - you could tell her anything or ask her anything and she’d be on your side.”* [Participant 2, 65 years old, Customer Service Role, Large Public Sector Organisation].

*“She said, ‘You’re valuable to us, you are not going anywhere, you are going to stay here.’ She knew when I’d gone for these other jobs and how disappointed I was and she said, ‘Well, their loss is our gain.’ We became better friends, and she supported me and I’ve had that throughout the last few years of my working in this department.”* [Participant 3, 64 years old, Customer Service Role, Large Public Sector Organisation].

*“Funnily enough, the management there, they love me. That’s what, they do not want me to leave, even though I’m 80, they do not want me to leave. They want me to stay.”* [Participant 7, 80 years old, Customer Service Role, Private Sector Retail Organisation].

(iii) Rejecting the label/stereotype as ‘older’ as a facilitator to wellbeing

A range of women described not feeling ‘older’ and not seeing their age as a negative factor whilst acknowledging the fact of ageing and the impacts physically and their age impacting on how they are perceived in work. Participant 10 exemplifies this in her description of only feeling old when she recently became a grandmother but also describing her doing activities that people of younger ages would be perceived to do like going to gigs. She described not feeling any different to younger colleagues as did Participant 7 who talked about feeling the same as other colleagues. However, Participant 7 also relayed *‘as you are getting to this age, I mean, especially on your feet all day. It starts to get too much. It’s all right if you are 60, and even 70, but once you are getting 80.’* She described the necessity to work financially and her intention to stop working soon. Participant 9 also described not thinking about ‘age’ but there being a fact of age and subtle ageism associated with it and the act of not thinking about her age seemed to allow her to feel more positive about her work despite feeling that people might be factoring her out.

*“As a woman over 60, I mean you reach the milestones, do not you, of age, and 60, 65 historically what I always thought of being pension age, which it used to be. Sixty-six is when I formally get my state pension and I cannot comprehend that really, to be honest. I do not feel any different. I’ve got aches and pains, yes, and I’ve not perhaps got as much stamina, but I try and keep myself fit… So, I’ve learnt some canny techniques to protect. I became a grandmother four years ago now and that was the first time I ever thought I was getting older. Certainly not in the workplace I do not feel old. It’s never mentioned either and I’ve got friends that are younger than me. Age is never a thing. I still go to gigs. I love live music. That perception of being old, it’s never changed what I do. Maybe in ten years’ time when I cannot stand up at a gig or whatever, I might feel it then. I’m sure there will become a time like that, but yes, at the moment, I do not feel any different.”* [Participant 10, 65 years old, Education Role, Large Public Sector Organisation].

*“I tend to not talk about age very much. I always think I will not do that, because I just think …*

*I do think there’s a huge amount of subtle discrimination that goes on in that sort of way about they are past it, they cannot keep up, and all that kind of thing”* [Participant 9, 64 years old, Social Care Role, Large Public Sector Organisation].

## Discussion

The current study aimed to explore the barriers and facilitators to workplace wellbeing in women aged 60 and over, with a focus on lower-paid roles, although a number of participants were also employed in senior positions. The taboo surrounding women’s wellbeing was evident across the interviews, echoing previous research (Grandey et al., 2020). Participants described the impact of women’s health issues spanning menopause, musculoskeletal conditions, and their consequences for wellbeing. The impact of women’s health issues spanning the menopause, musculoskeletal issues, and the impact on wellbeing was apparent, mirroring and building upon previous findings and evidencing the long-term impact of women’s health issues across the “Three M’s” (Brown et al., 2024; Heikkinen et al., 2022). In addition, there was an overwhelming burden of caring and responsibilities for management and organization of the household, relayed by women across the interviews, that was echoed as a negative impact of being a woman in work on their wellbeing. Miller et al. (2022) showed that older women have significantly more stress accumulations across the life course, negatively impacting their health and wellbeing, and these findings would suggest that these burdens are a significant factor in this trend.

Warr’s (2011) evidence that equity is a ‘vitamin’ for wellbeing in work would suggest inequitable practices for women would negatively impact on wellbeing. Women across the interviews felt that more should be done to accommodate their needs in the workforce in terms of workplace accommodations and flexible working and that negative impacts of being a woman going through these stages included a lack of promotion and impacts on confidence could be prevented. This also builds upon factors for wellbeing identified by Warr (2007) in respect of career outlook and valued social position or perception of significance because women often feel undervalued and overlooked. This adds to previous studies, such as Hickey et al. (2017), who found that women perceive that the menopause is perceived negatively in the workplace and that male ideal worker norms are preferred (Padavic et al., 2020; Steffan, 2021), in contrast to those that embody femininity (the taboo of the Three M’s). In addition, the impact of the pension changes in the UK meant that women were negatively impacted emotionally and psychologically, facing the necessity to work for longer. As highlighted in the introduction, the UK women in the 55–64 years old group show the highest levels of sickness absence (Office for National Statistics (ONS), 2024).

The current study highlighted promoting the value of older women in work and emphasizing their importance considering intergenerational barriers would act as a facilitator to wellbeing and negative perceptions of growing older. This relates to social identity complexity theory (Roccas and Brewer, 2002) and is associated but distinct from stereotype threats whereby ageing women are shown to perform better when the stereotype about older women is removed (Manzi et al., 2021). This seems particularly important regarding the theme around intergenerational barriers and perceptions of a digital age divide. Women discussed rejecting the label of being older and highlighted their value in tandem with emphasizing the perception that they were seen as less ‘tech savvy’ and competent than ‘younger colleagues’, highlighting a clear dissonance in the construction of their older worker identities. Positive factors to support women’s wellbeing included support from line management and the community and social elements of work, which is echoed in the literature (Warr, 2007).

The findings also emphasize a feeling of invisibility women, in both lower-paid and more senior roles, typified by feeling unseen and unheard compared to younger workers and feeling to be seen as less competent, including within the technology age divide, which supports previous literature highlighted in the introduction (Barrett, 2022; Westwood, 2023; Riach, 2025). There is evidence that also points to technostress in older people, but this is a complex picture (Nimrod, 2018). There were also gendered elements and a feeling of their experience as an older woman not being valued, whilst women acknowledged the value and ‘kudos’ they bring. Whilst the perception of older people having techno-anxiety element is not necessarily gendered, importantly, an integrative review highlighted that whilst technostress and anxiety appear not to be clearly evidenced in older workers, suggesting a disconnect and a stereotype in the workplace, there was a need identified to explore intersectional impacts, such as with age and gender (Nedeljko et al., 2024). Gendered elements to these findings center around the roles that women tend to adopt (5Cs: ‘catering, cleaning, caring, clerical, and cashiering roles’) and the patronizing notion of being seen as a ‘grandma’ figure who was slow with technology and more suited to being a “tea lady” or more domestic roles.

Valuing older women in work was seen as a facilitator to wellbeing as was support from line management and rejecting the label/stereotype of being ‘older’, which echoes Taylor et al.’s (2021) work. This builds upon previous ideas around the notion of ageing as a performance and the idea of reconceptualising ageing and how societies think about ageing as part of an ‘evergreen’ agenda so that we see ageing in a positive light (Barrett, 2022; Scott, 2024). The need to focus on women specifically has also been highlighted because of the proportion of women in the age group 55 and over projected in the coming years as well as a lack of consideration for gendered ageing elements in extended working life policy in the UK (Organisation for Economic Co-operation and Development (OECD), 2024; Ni Léime et al., 2017).

There are several limitations within the present study. First, that the sample was limited to the Northwest of England and may not be transferable to some roles, e.g., highly skilled roles, although there are transferable elements. Second, the sample lacked diversity in respect of ethnicity, and future research could use an intersectional lens. However, the study also has several strengths in focusing on the intersection of ageing, gender, and those in lower-paid roles. Future research should focus on the intersectional experiences of women and focus on the life-course accumulations of women’s needs in the workforce and the factors that can support women as they age in work, particularly with advances in technology. For instance, in a larger study, it would be beneficial to collect data on the respondents’ lifelong caring responsibilities, domestic circumstances, and their pay and pension status.

### Implications for policy and practice

There are several implications for policy and practice in terms of workplace interventions to support women’s wellbeing as they age. Policies to support women’s health and wellbeing should focus on reversing the taboo of women’s wellbeing and translating policy into action to support women in having conversations about lifelong wellbeing, e.g., menopause and workplaces adapting to women’s needs with flexibility across the life course to support women’s wellbeing. Valuing older women in work and support from line management were key facilitators to wellbeing, but there was a clear taboo of womanhood felt by women, as well as a feeling of being unheard and invisible. Workplaces need to promote a culture of support and play an active role in reversing the taboo of women’s wellbeing and the stereotype of ageing intersected with gender.

## Data Availability

The datasets presented in this article are not readily available because not open access. Requests to access the datasets should be directed to c.e.edge1@salford.ac.uk.
